# Age-specific changes in genome-wide methylation enrich for Foxa2 and estrogen receptor alpha binding sites

**DOI:** 10.1371/journal.pone.0203147

**Published:** 2018-09-26

**Authors:** Nishanth Uli, Eduardo Michelen-Gomez, Enrique I. Ramos, Todd E. Druley

**Affiliations:** 1 Center for Genome Sciences and Systems Biology, Washington University School of Medicine, St. Louis, Missouri, United States of America; 2 Department of Pediatrics, Washington University School of Medicine, St. Louis, Missouri, United States of America; 3 Department of Genetics, Washington University School of Medicine, St. Louis, Missouri, United States of America; 4 Department of Developmental Biology, Washington University School of Medicine, St. Louis, Missouri, United States of America; University of Texas Rio Grande Valley, UNITED STATES

## Abstract

The role of DNA methylation patterns in complex phenotypes remains unclear. To explore this question, we adapted our methods for rare variant analysis to characterize genome-wide murine DNA hybridization array to investigate methylation at CpG islands, shores, and regulatory elements. We have applied this platform to compare age and tissue- specific methylation differences in the brain and spleen of young and aged mice. As expected from prior studies, there are clear global differences in organ-specific, but not age-specific, methylation due mostly to changes at repetitive elements. Surprisingly, out of 200,000 loci there were only 946 differentially methylated cytosines (DMCs) between young and old samples (529 hypermethylated, 417 hypomethylated in aged mice) compared to thousands of tissue-specific DMCs. Hypermethylated loci were clustered around the promoter region of *Sfi1*, exon 2 of *Slc11a2*, *Drg1*, *Esr1* and *Foxa2* transcription factor binding sites. In particular, there were 75 hypermethylated Foxa2 binding sites across a 2.7 Mb region of chromosome 11. Hypomethylated loci were clustered around *Mid1*, *Isoc2b* and genome-wide loci with binding sites for Foxa2 and Esr1, which are known to play important roles in development and aging. These data suggest discreet tissue-independent methylation changes associated with aging processes such as cell division (*Sfi1*, *Mid1*), energy production (*Drg1*, *Isoc2b*) and cell death (*Foxa2*, *Esr1*).

## Introduction

Aberrations in the processes that regulate healthy aging result in a wide array of disease and disability. However, the molecular, genetic and epigenetic regulation of these processes is only moderately understood. Epigenetic modifications (including DNA methylation) are heritable but potentially reversible and may change throughout life. Methylation of cytosine residues represents an important epigenetic mechanism that typically acts to repress gene transcription. The link between aging and DNA methylation levels has been extensively studied, and recent studies have looked at analyzing methylation as a proxy for the aging process as a whole[1,2]. DNA methylation can be affected by different factors such as environment, lifestyle, diet and toxin exposure[3,4], and has been shown to play an important role in complex disease such as cancer[5–9], metabolic disorders [10,11], neurological diseases[12–14], cardiovascular diseases[15,16] and myopathies[17,18]. Further, changes in DNA methylation have been shown to play a role in learning, memory, and aging-associated cognitive decline[19–22].

Previously, *global* levels of DNA methylation have been shown to decrease with aging[7,23–25]. However, most studies used relatively low resolution array-based platforms that mainly focus on CpG islands and repetitive elements, neglecting other regulatory elements in the genome[24–26]. Because of this, the overall loss of methylation is attributed to non-functional LINE and Alu sequences, rather than functional genomic regions[27,28]. To the contrary, several studies have demonstrated age-specific *hyper*methylation at bivalent chromatin domains[29,30] containing developmental and regulatory loci[31] such as the polycomb repressor complex[32], estrogen receptors[33–35], *IGF2*[36] and many others.

In order to characterize age-specific, genome-wide DNA methylation changes outside of CpG islands and repetitive elements, we employed a targeted, genome-wide murine methylome capture array followed by a high-throughput bioinformatic workflow to efficiently and accurately survey discreet variations in DNA methylation. As proof of principal, we focused on comparisons between brain and spleen methylation in juvenile (1–2 month) and aged (>20 months) mice. The rationale was that brain tissues have low levels of cell division and environmental exposure due to the blood-brain barrier compared to the highly replicating tissues of the reticuloendothelial system and their exposure to anything in the bloodstream.

## Materials and methods

### Designing the RNA hybridization “bait” library

The Agilent SurePrint oligo manufacturing and SureSelect hybridization target enrichment technology uses biotinylated 120 base pair RNA “baits” to selectively hybridize with complementary sequences in a sample of randomly fragmented source DNA. In collaboration with Agilent, we designed a murine-specific DNA methylation hybridization array that targets up to 84 Mb of CpG islands, shores, shelves and regulatory elements in the mouse genome based on criteria described below. Using databases from the National Center for Biotechnology Information (NCBI-NIH) (http://www.ncbi.nlm.nih.gov), the University of California Santa Cruz (UCSC) Genome Browser (http://genome.ucsc.edu) and Ensembl (European Bioinformatics Institute/Wellcome Trust Sanger Institute; http://www.ensembl.org), we obtained the genomic coordinates for all known CpG islands, shores and shelves in the mouse genome (mm9). Additionally, we included tissue-specific differentially methylated regions (DMR)[37], DNase I hypersensitive sites, and different regulatory elements such as promoters, enhancers and transcription factor binding sites (S1 Table). The combined total mouse methylome design included ~201,788 targets and ~110Mb of bases covered (due to some overlap between target regions and regions of systematically poor hybridization, the final region covered is ~85-90Mb). The on-target enrichment for each sample at each of the categories included in the target design is found in the Supporting Information (S2 Table). This product is commercially available from Agilent (catalog number 931052) and has been used to identify methylation patterns associated with murine neurodevelopment [38].

### Mice

Harvested brains and spleens from three juvenile (1–2 months) and three aged (>20 months) C57/BL6 mice were kindly provided by Dr. James DiGregori at the University of Colorado—Denver. All three biological replicates were prepared and sequenced independently for the methyl-seq experiments described.

### DNA extraction

Extraction of DNA was performed using Qiagen DNeasy Blood & Tissue extraction kit (catalog number 69504) as described in the manufacturer’s protocol. The DNA yield from the extraction resulted in a range from 7–15μg per sample and was quantified using Molecular Probes fluorescent SYBR Gold (catalog number S11494) assay as previously described by Druley *et al* [39].

### Methyl-seq library preparation and bisulfite treatment

Methyl-Seq libraries were prepared using Agilent SureSelectXT Mouse Methyl-Seq (catalog number 931052) and SureSelectXT Reagent Kit, HSQ (catalog number G9611A) as described in the manufacturer’s protocol. Hybridization, recovery and enrichment were done according to protocol Version A, August 2012 with no modifications. A total of 3μg of DNA was used for the library preparation. Following hybridization capture, bisulfite treatment converted unmethylated cytosines to uracils using the Zymo Research, Zymo EZ DNA Methylation-Gold (catalog number D5005) kit following manufacturer’s protocol.

### Sequencing

Next-generation sequencing was performed in the Edison Family Center for Genome Sciences and Systems Biology at Washington University using the Illumina HiSeq 2500 platform generating 101bp paired-end reads, a total of 3 samples were multiplexed for each sequencing lane generating approximately 90–100 million reads per sample (S2 Table).

### Data analysis and bioinformatics

Sequence data was visualized on the Washington University Epigenome Browser (http://epigenomegateway.wustl.edu/) via a methylC track that displays read depth and methylation score for every locus of interest across the genome. The Bismark-generated SAM files for each sample were converted to BAM files using the Samtools view function. The resulting BAM files were then sorted and indexed using Samtools. The methylation extractor included as part of the Bismark suite was used to generate a bedGraph file that included data on methylation percentage and read depth for all cytosines in a CpG context, using the options -p—comprehensive—bedGraph—zero_based. The methylation score and read depth at each locus were separated and written into their own files, resulting in two bedGraph files for each sample: one that contained the methylation score at each locus, and another that contained the corresponding read depth at that locus. The Bismark methylation extractor was also used to generate a M-bias plot for each sample. The bedGraph files were then zipped via bgzip and then indexed via tabix. The files were uploaded to a web server and submitted on the Epigenome Browser to allow for data visualization.

The R package “methylkit” was used to determine DMCs. The Bismark-generated SAM files were sorted by chromosome position and read into methylKit using the ‘processBismarkAln’ function in order to create methylKit-compatible call files for each sample. Using the ‘getMethylationStats’ function, a histogram for percent methylation distribution was obtained for each sample. Using the ‘filterByCoverage'function, the samples were filtered by read coverage, discarding bases that had less than 5x read coverage. Each sample was then tagged with a treatment condition based on both age (young vs. aged) and tissue type (brain vs. spleen). Samples were then clustered using the ‘clusterSamples’ function, which hierarchically clusters samples together based on the similarity of their methylation profiles and draws a dendrogram illustrating this similarity. A PCA Analysis was then performed on the samples using the ‘PCASamples’ function, which uses the R function ‘prcomp’ to perform a principal components analysis on the samples using percent methylation matrix as an input. Using the ‘calculateDiffMeth’ function, DMCs were then obtained for the following comparisons: young brain vs. old brain, young spleen vs. old spleen, young brain vs. young spleen, old brain vs. old spleen, young vs. old independent of tissue type, and brain vs. spleen independent of age. These DMCs were obtained by assigning each sample to a particular treatment condition and then comparing two different treatment conditions using the ‘calculateDiffMeth’ function, which uses logistic regression to calculate q-values for differential methylation between two treatment types. For example, in order to determine age-specific DMCs independent of tissue type, the three young spleen samples and three young brain samples were pooled together by being given the same treatment condition: “young”. The three old brain and three old spleen samples were also given their own treatment condition (“old”), and the DMCs between these two treatment conditions were located by ‘calculateDiffMeth’. After the calculation of q-values and methylation levels by ‘calculateDiffMeth’, the DMCs were then filtered based on methylation score difference between treatments and q-value using the ‘getMethylDiff’ function. Only bases that had a q-value <0.05 and methylation difference >25% between the two treatment conditions were selected.

In order to annotate these filtered DMCs, BED files containing annotation data for RefSeq genes, CpG locations and ORegAnno data for the mm9 genome were downloaded from the UCSC Genome Browser. The DMCs for each comparison were then annotated in methylKit using the ‘annotateWithGenicParts’ and ‘annotateWithFeatureFlank’functions, which use downloaded annotation data to specifically mark up DMCs. Pie graphs indicating the percentage of bases that overlapped with genic features (promoter, exon, intron, and intergenic) and CpG features (in shores, shelves or other) were obtained for each comparison using the ‘plotTargetAnnotation’ function. In order to better visualize the age-specific and tissue-independent DMCs, the DMC locations obtained using ‘methylKit’ were used to guide localization of these same regions on the Epigenome Browser, where they could be visually interpreted to supplement our quantitative interpretation. DMCs that overlapped significantly with important genic and regulatory features could then be properly visualized and quantitated. All sequencing data generated and analyzed in this study are available at the NCBI Sequence Read Archive as follows: young brain at SAMN09911432, young spleen at SAMN09911433, aged brain at SAMN09911434, aged spleen at SAMN09911435.

### Reduced representation bisulfite sequencing

Validation of called DMCs was performed using the reduce representation bisulfite sequencing (RRBS) pipeline as described by Schillebeeckx *et al* [40]. Briefly, sequencing reads were aligned to the mouse genome (build mm9/ NCBI37) reference using RRBSMAP[41] filtering against reads that contain adapter sequence. Reads that showed less than 90% bisulfite conversion (~1 unconverted non-CpG cytosine per read) were filtered to remove those that resulted from incomplete bisulfite converted molecules. Aligned reads with a mapping quality of zero were also discarded. The resulting high quality uniquely mapped reads were used for methylation calling. Genomic coordinates of all CpGs in the reference sequence were identified and assessed percent DNA methylation by calculating the fraction of reads that had an unconverted cytosine at the CpG position relative to the total reads. Each read requires to have either a “TG” or “CG” dinucleotide at the expected CpG coordinate to be considered for analysis. DMCs were determined using a Fisher’s Exact Test comparing the ratio of methylated versus unmethylated reads for each sample. CpGs were considered hypomethylated or hypermethylated if they showed a statistically significant decrease or increase of at least 25% [40], respectively.

## Results

### Target capture and bioinformatic metrics

After analyzing 90-100M reads per sample, we found ~81.5% of raw reads align to target and ~85% are on-target. For each sample, ~90% [~90 Mb] of the targeted methylome was captured and sequenced with a mean target coverage greater than 50X (S2 Table). The remaining 9 Mb of non-captured targets are uniformly absent across multiple samples indicating a systematic failure of hybridization capture at these loci.

### Global levels of DNA methylation

Contrary to prior reports and because our targets were not enriched for repetitive elements, we did not observe a substantial decrease in overall methylation levels as a function of age. The percentage of age-specific levels of methylation is approximately equivalent for spleen [42.97% young vs. 43.23% aged] and brain [39.53% young vs. 37.10% aged].

### Tissue-specific methylation differences

**Fig 1:**
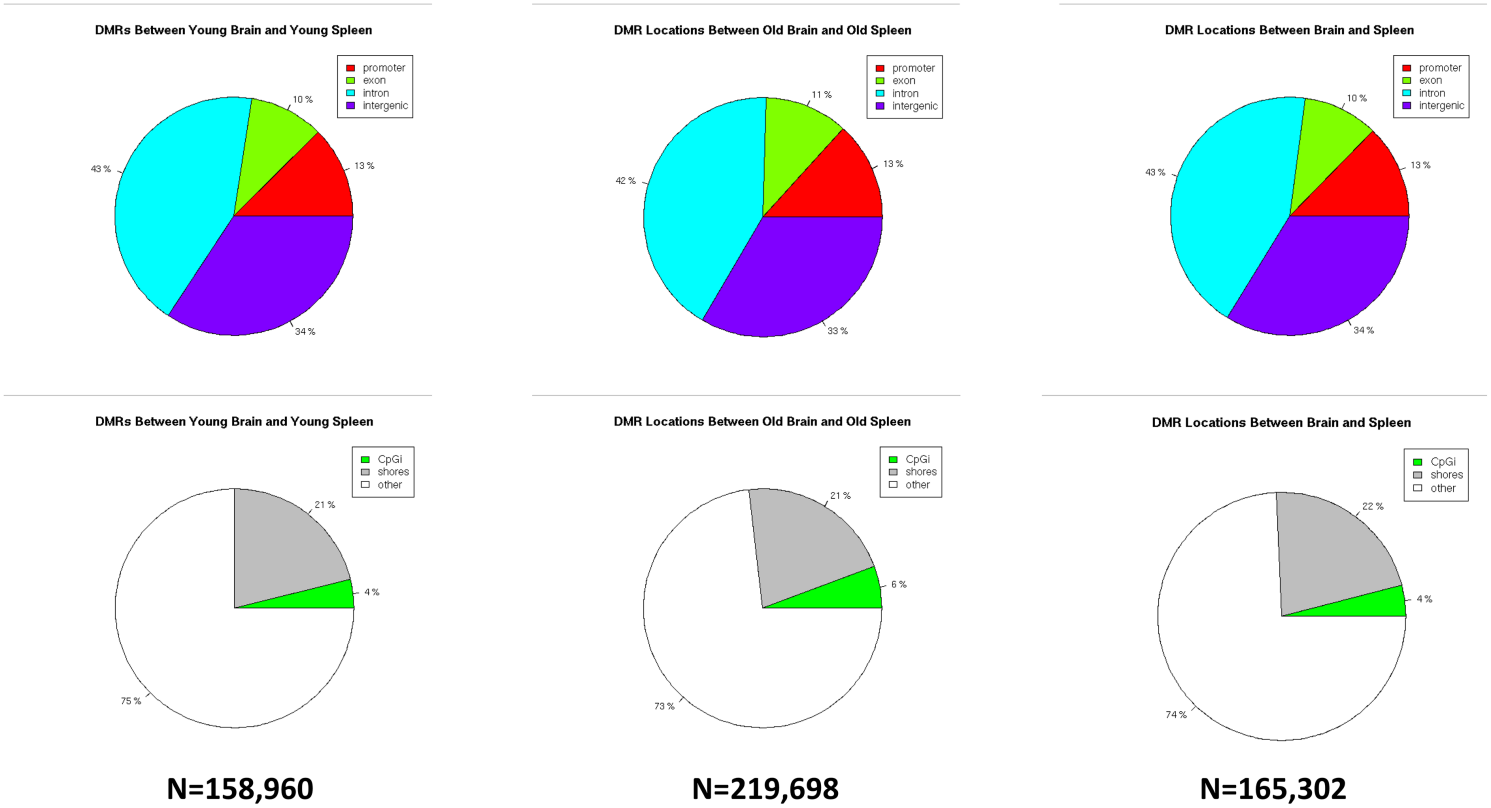
Tissue-specific differentially methylated cytosines. The percent breakdown of locations for differentially methylated cytosines [DMCs] as a function of tissue type between young brain and young spleen [left column], between old brain and old spleen [center column] and a pool of all brain vs all spleen irrespective of age [N = 12 total in the right column]. The top row delineates between DMCs located at promoters, exons, introns or intergenic regions. The bottom row delineates between DMCs located at CpG islands [CpGi], CpG shores or other regulatory regions (see S1 Table).

Separating methylated regions into subclasses [i.e. DMCs, regulatory elements, CpG islands, shores and shelves] also shows no significant age-specific differences in overall DNA methylation levels. In contrast and as expected, when comparing the same subclasses between brain and spleen, significant differences were noted in overall DNA methylation. When comparing tissue-specific differences between spleen and brain independent of age, we identified 165,302 DMCs. These DMCs were predominantly located in non-CpG island and shore regions, which were mostly located within intronic and intergenic regions (Fig 1).

### Age-specific methylation differences

**Fig 2:**
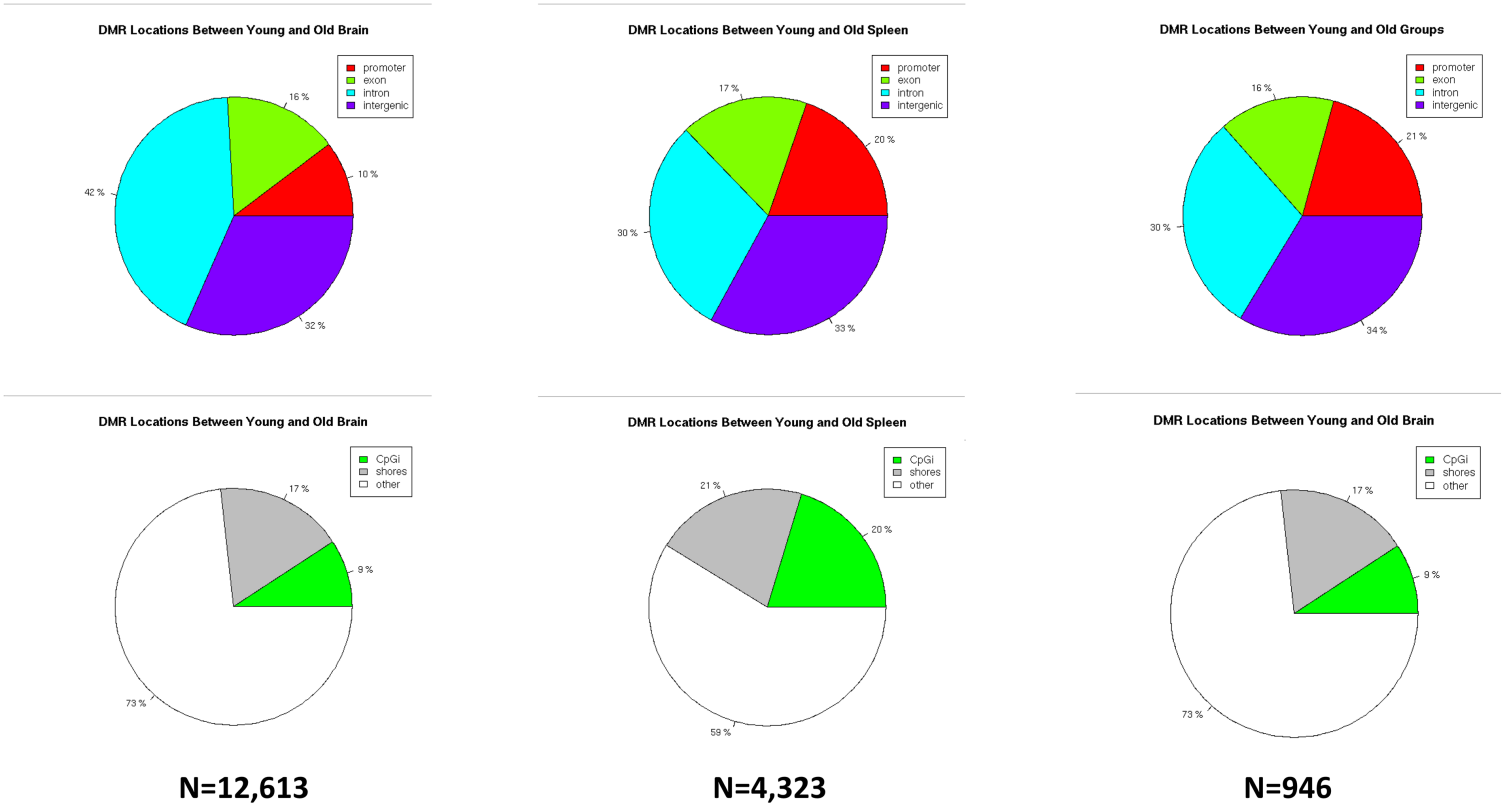
Age-specific differentially methylated cytosines. The percent breakdown of locations for differentially methylated regions [DMCs] as a function of age between young and old brain [left column], between young and old spleen [center column] and an aggregate of all young versus all old samples [N = 12 total in the right column]. The top row delineates between DMCs located at promoters, exons, introns or intergenic regions. The bottom row delineates between DMCs located at CpG islands [CpGi], CpG shores or other regulatory regions (see S1 Table).

**Fig 3:**
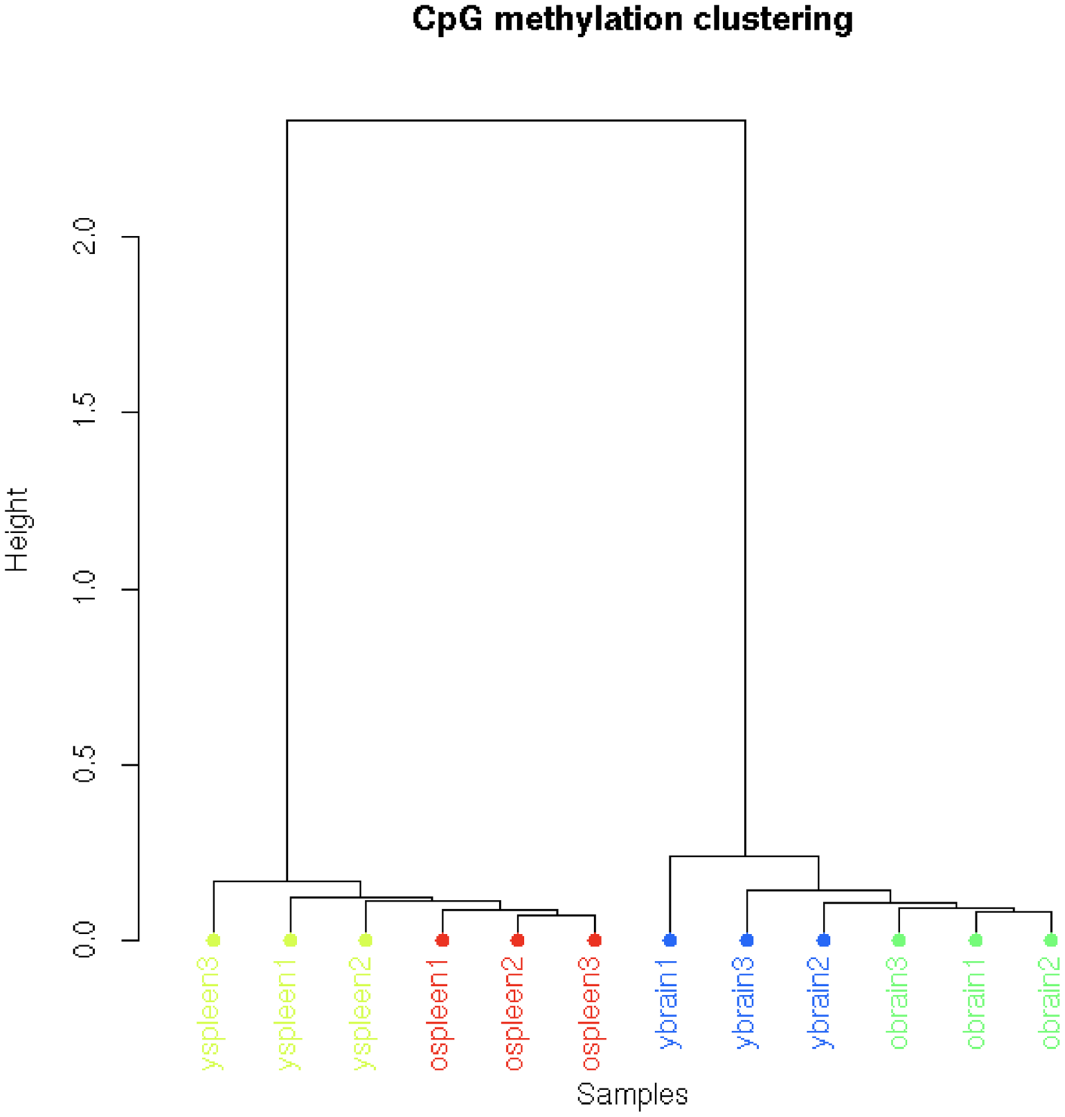
Dendrogram of individual tissue sample clustering based on patterns of CpG methylation. When clustering the 12 samples by DMCs, the large number of tissue-specific versus age-specific DMCs is driving the principal component analysis [S1 Fig] and this clustering dendrogram showing that, when clustering by methylation similarities, samples cluster much more tightly as a function of tissue type rather than a function of age.

When examining differences between young and aged groups, we identified only a few thousand loci that had significant differences in methylation (Fig 2). By examining the overlap between brain and spleen at these loci, we identified 946 loci that were differentially methylated as a function of age independent of tissue. Of these, 529 were hypermethylated in aged mice and the remaining 417 were hypomethylated. We found that [spleen and brain] age-specific DMCs were significantly more likely to be found in CpG island/shores and in exonic/promoter regions of the genome compared to the tissue-specific DMCs [p = 0.024, one tailed t-test]. The large number of tissue-specific, versus age-specific, DMCs is also driving the PCA analysis and clustering dendrogram showing that, when clustering by methylation similarities, samples cluster much more tightly as a function of tissue type rather than a function of age (Fig 3, S1 Fig).

### Validation using RRBS

Methylation calls from target capture were validated using the orthogonal platform, RRBS [40]. Methylation calls within each group [young brain, aged brain, young spleen, aged spleen] were compared against RRBS throughout the mouse genome. Looking at a range of 1,397–3,266 sites we found on average a concordance of approximately ~75% [range 69.6%-76.9%] between the two different platforms. The strong concordance between methods suggested that our sequencing results for these heterogeneous methylation signals were accurate.

### Hypermethylated DMCs in aged mice

**Fig 4:**
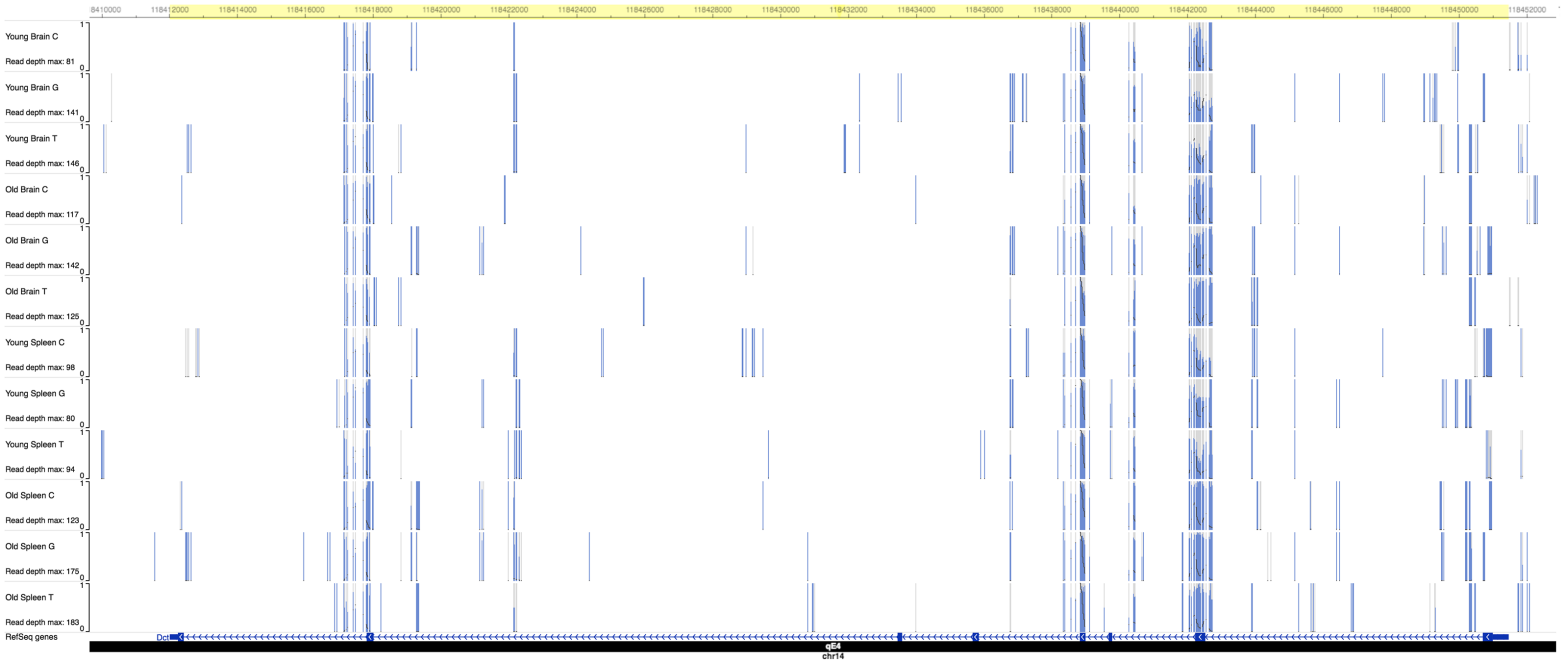
Aged mouse-specific hypermethylation of exon 2 of *Dct*. An Epigenome Browser [http://epigenomegateway.wustl.edu/] image of the cluster of differential methylation on chromosome 14 over exon 2 of the *Dct* gene between bases 118,442,000 and 118,443,000 [mm9]. Within the highlighted region, the specimens from old mice demonstrate an excess of methylation [height of darker blue bars] compared to the young mice.

**Fig 5:**
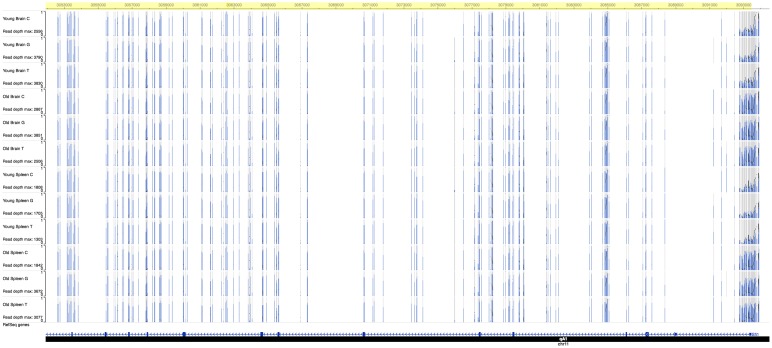
Aged mouse-specific hypermethylation of *Foxa2* binding sites at the transcription start site of *Sfi1*. An Epigenome Browser image of the cluster of differential methylation on chromosome 11 over the transcription start site of the *Sfi1* gene between bases 3,092,600 [approximate] and 3,094,000 [mm9]. Within the highlighted region, the specimens from old mice demonstrate an excess of methylation [height of darker blue bars] compared to the young mice. This region contains several Foxa2 binding sites and is adjacent to three known regulatory domains: OREG1667474, OREG1756550, and OREG0042551.

Bioinformatic analysis and visualization of the 529 hypermethylated DMCs on the Washington University Epigenome Browser allowed localization of age-specific DMCs. In aged mice, we found a clustering of hypermethylated DMCs at exon 2 of the *Slc11a2 [Dct]* gene on chromosome 14 (Fig 4), and at the transcription start sites of *Sfi1* and *Drg1*. We also noted a preponderance of hypermethylation at binding sites for the pioneer factor *Foxa2* and transcription factor *Esr1*. There were 14 different regulatory sites for *Foxa2* throughout the genome that contained at least one hypermethylated DMC. One of these regulatory sites on chromosome 11 [OREG1667474] showed 75 discreet sites of hypermethylation across 2.7 Mb overlying the 5’ end of the *Sfi1* open reading frame (Fig 5), which was also adjacent to two other regulatory domains, OREG1756550 and OREG0042551. There were four such hypermethylated sites for *Esr1* across chromosomes 6, 8, and 17. Of note, the hypermethylated *Foxa2* binding sites were primarily found on chromosome 11 as well as the hypermethylation at *Sfi1* and *Drg1*.

### Hypomethylated DMCs in aged mice

DMCs with a significant lack of methylation in aged mice were observed clustered within a 2Mb regulatory regions on the X chromosome [OREG1829350 and OREG0052202] within 2,708 bp of each other, at the end of the *Mid1* gene, as well as the transcription start site of *Isoc2b* on chromosome 7. In addition to the hypermethylated regions described above, we identified multiple hypomethylated regulatory regions for *Foxa2* and *Esr1* as well. The OREG0052202 regulatory site on the X chromosome harbors 8 Foxa2 binding sites. There were 13 additional regulatory sites across the genome for *Foxa2* that showed at least one hypomethylated site. There were 8 regulatory regions for *Esr1* with at least one hypomethylated DMC. These were by far the most common TFBS associated with the DMCs we identified.

## Discussion

Here, we implemented a genome-wide hybridization capture method to query large portions of the murine methylome. We chose to use this hybridization strategy because it is more cost-effective in terms of sequencing and computational analysis than whole genome bisulfite sequencing, but is more comprehensive for regions of differential methylation than microarrays, which cannot offer single base resolution. Other methods such as MeDip-Seq, methylation-sensitive restriction enzymes [MSRE] and RRBS cannot be targeted, which limits their utility.

In this study, we interrogated differences in DNA methylation as a function of age between two murine tissues [brain and spleen] that have very different cellular proliferation rates and are functionally/structurally diverse. Indeed, the spleen in particular has an important role as a integral part of the immune system, and we have known for some time that both adaptive and innate immune cells experience drastic functional alterations as a result of the aging process [42–44]. Aging, then, represents a valuable window through which to capture changes that have both physiologic and pathologic consequences, not just in the immune system but across the body as well. Through this comparison, our goal was to identify regions of age-specific differential methylation that were consistent between each tissue, suggesting a more universal role in the aging process.

Unlike prior observations, we find no significant change in the overall global levels of age-specific DNA methylation. We believe this difference is due to the fact that most prior studies have used methylation survey methods that mainly focused on CpG islands and transposable repetitive elements [e.g. Alu and L1][27,28]. The targeted methylome hybridization array avoids repetitive element sequence as targets and integrates many genomic regions that are typically absent in arrays, MeDIP, RRBS or MSRE methods, which allows us to compare putative regulatory targets in greater detail.

While descriptive in nature, we identified an age-specific enrichment of differential methylation in binding sites for the transcription factors *Foxa2* and *Esr1*, which are known to play important roles in development and aging[33,45–48]. *Foxa2* belongs to the family of forkhead box transcription factors known to regulate multiple stages of mammalian life, beginning with early development and continuing during organogenesis[45]. FoxA proteins are considered ‘pioneer factors’ that can engage silent chromatin and initiate a cascade of gene regulation, networks of subsequent transcription factors—so called batteries and terminal selector genes—resulting in the precise specification of a given cell or tissue type[49]. Our results are consistent with work showing that hypermethylation of *Foxa2* promoters and binding sites corresponds with premature and replicative senescence[50], hallmarks of disease-associated and physiologic aging, respectively. In contrast, *Esr1* encodes the estrogen receptor-alpha (ERalpha] protein, which acts as a ligand-activated transcription factor. Age-specific hypermethylation of *ESR1* promoters and downstream effectors has been described in colon[35], prostate[48] and hypothalamus[34]. There is additional evidence linking this receptor to the regulation of genes previously known to be involved in aging such a: *Ep300*, *Sp1*, *Src*, *Tff1*, *Ccnd1*, *Ncoa1*, *Ncoa2* and *Ncoa3*[34,47,51,52]. Strikingly, Carroll et al. demonstrated that 54% of estrogen receptor binding regions also contain Forkhead factor binding sites and that FOXA1 co-binding was necessary for ERalpha-mediated activation of estrogen receptor target genes on human chromosomes 21 and 22 in MCF-7 breast cancer cells stimulated by estrogen[53]. Much of human chromosome 22 is homologous to mouse chromosome 11, which contains the hypermethylated regulatory sequences and genes in aged mice found in our survey. Mouse chromosome 11 has previously been implicated in premature death, infertility, runting and neurological defects[54]. Nearby genes on chromosome 11 with hypermethylation at transcription start sites were *Sfi1*, which is a spindle protein important for mitosis and organelle biogenesis[55] and *Drg1* [developmentally regulated GTP-binding protein 1], which is a highly conserved gene important for development and regulating cell growth and acts as a tumor suppressor[56]. The specific hypermethylation of TSSs at these two genes could point to age-related transcriptional repression of these two important genes involved in cell division and energy production, respectively. The identification of a region of hypermethylation at exon 2 of the *Dct* [Dopachrome tautomerase or *Trp2*] gene is more curious as this gene is well known for regulation of melanin production and reactive oxygen species[57], but not associated with longevity. However, it is an assumption that the gene[s] controlled by differential methylation are those in closest proximity. In fact, FOXA1/ERalpha binding was shown to act on enhancers that influenced expression of target genes as much as 100 kb away from the binding site[53], so the downstream effects of these age-specific differentially methylated regions on mouse chromosomes 11 and X may be quite distant and far ranging. We also found discrete areas of age-related hypomethylation on chromosome 7 at the transcription start site of *Isoc2b*, an isochorismatase-like protein, and two sites on the X chromosome near the end of the *Mid1* gene, which encodes a microtubule-binding protein important for cell division, and whose overexpression has been implicated in the pathogenesis of Opitz syndrome[58]. Age-related hypomethylation of these sites could point to transcription-level increases in expression as part of the aging process that could have physiologic and pathologic implications, which represents an interesting avenue for future research. A limitation of our study is that we do not have correlative gene expression data from our tissues or in public repositories as gene expression studies from mice aged >20 months is not common.

## Conclusions

Using a novel murine hybridization capture array, we were able to characterize genome-wide changes in DNA methylation as a function of both tissue type and age. By expanding our genomic analysis beyond CpG islands and repetitive islands, we were able to accurately survey global differences in methylation levels across the genome as well as discreet changes in individual genomic areas. Our analysis showed that unlike previous reports, there was no significant global decrease in methylation as a function of age. However, we were able to identify discreet areas of tissue and age-dependent differential methylation. In particular, we were able to identify areas of tissue-independent differential methylation in regions of the genome, both coding and regulatory, associated with mechanisms of aging. In summary, these results suggest that there are tissue-independent mechanisms of age-specific differential methylation that influence specific transcription factor binding and subsequent gene regulation. These data will facilitate hypothesis-driven research into the role of methylation on regulatory mechanisms involved in healthy aging.

## Supporting information

S1 Table Mouse methylome target capture design. This table outlines the inclusion of loci included in the murine methylome hybridization capture enrichment design.
(DOCX)

S2 Table Methyl-seq capture and bioinformatics metrics for spleen and brain samples. Metrics for the raw reads and percent enrichment for each specimen studied.
(DOCX)

S1 Fig Principal component analysis. Principal component analysis demonstrates clustering based on tissue type rather than age.
(DOCX)

S2 Fig The global methylation at CpG, CHG and CHH sites. The type and percent of methylation between young and aged samples is not different across the targeted methylome.
(DOCX)
