# Supplementary figures and images for: Age-specific changes in genome-wide methylation enrich for Foxa2 and estrogen receptor alpha binding sites
Source: PLoS One. 2018 Sep 26;13(9):e0203147. doi: 10.1371/journal.pone.0203147 (PMC6157835; doi:10.1371/journal.pone.0203147)

**Supporting Table 1 (S1 Table): Mouse methylome target capture design.**


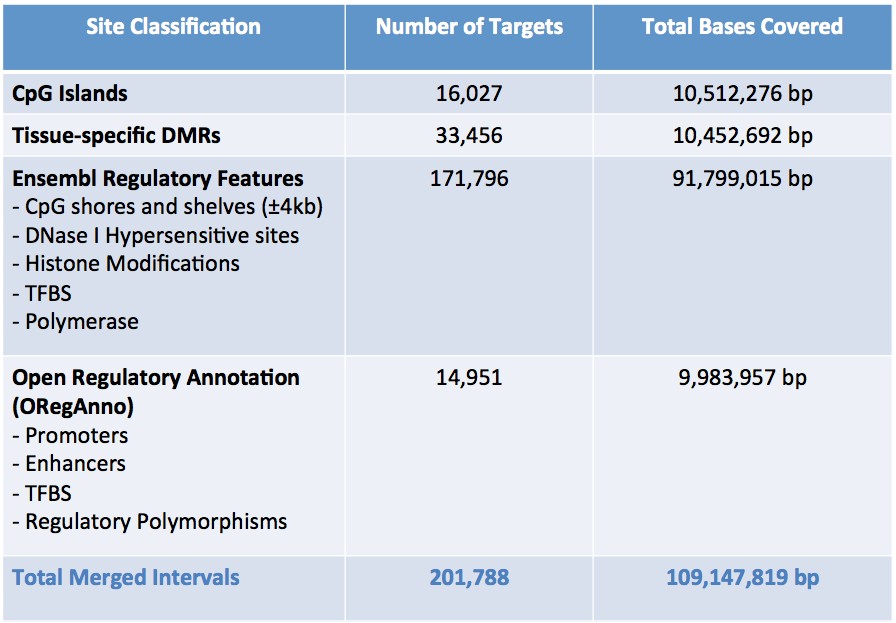

Supplement: S1 Table — This table outlines the inclusion of loci included in the murine methylome hybridization capture enrichment design. (DOCX) [file pone.0203147.s001.docx]
