# Supplementary material for: Age-specific changes in genome-wide methylation enrich for Foxa2 and estrogen receptor alpha binding sites
Source: PLoS One. 2018 Sep 26;13(9):e0203147. doi: 10.1371/journal.pone.0203147 (PMC6157835; doi:10.1371/journal.pone.0203147)

**Supplementary Table 2 (S2 Table): Methyl-seq capture and bioinformatics metrics for spleen and brain samples**


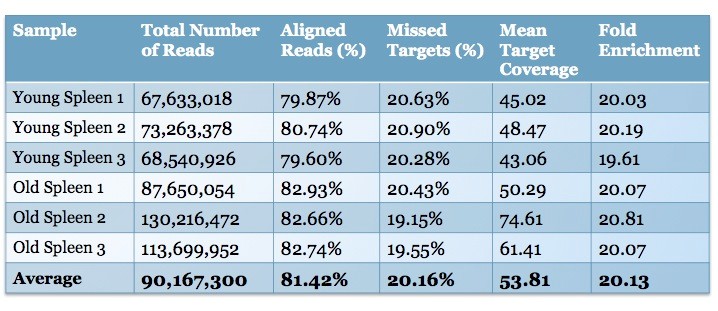


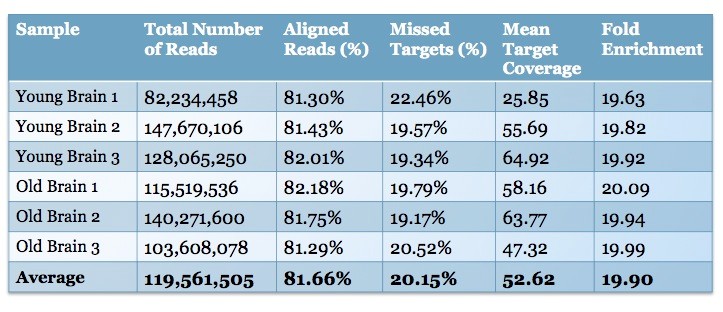

Supplement: S2 Table — Metrics for the raw reads and percent enrichment for each specimen studied. (DOCX) [file pone.0203147.s002.docx]
