# Supplementary material for: Age-specific changes in genome-wide methylation enrich for Foxa2 and estrogen receptor alpha binding sites
Source: PLoS One. 2018 Sep 26;13(9):e0203147. doi: 10.1371/journal.pone.0203147 (PMC6157835; doi:10.1371/journal.pone.0203147)
Supplement: S1 Fig — Principal component analysis demonstrates clustering based on tissue type rather than age. (DOCX) [file pone.0203147.s003.docx]

**Supplementary Figure 1 (S1 Fig). Principal component analysis.** Sequencing results generating sample-specific methylation profiles were clustered by principal component analysis according to differentially methylated cytosines (DMCs). As expected, given the large number of tissue-specific DMCs, the tissue types clustered quite distinctly with less resolution due to age. **
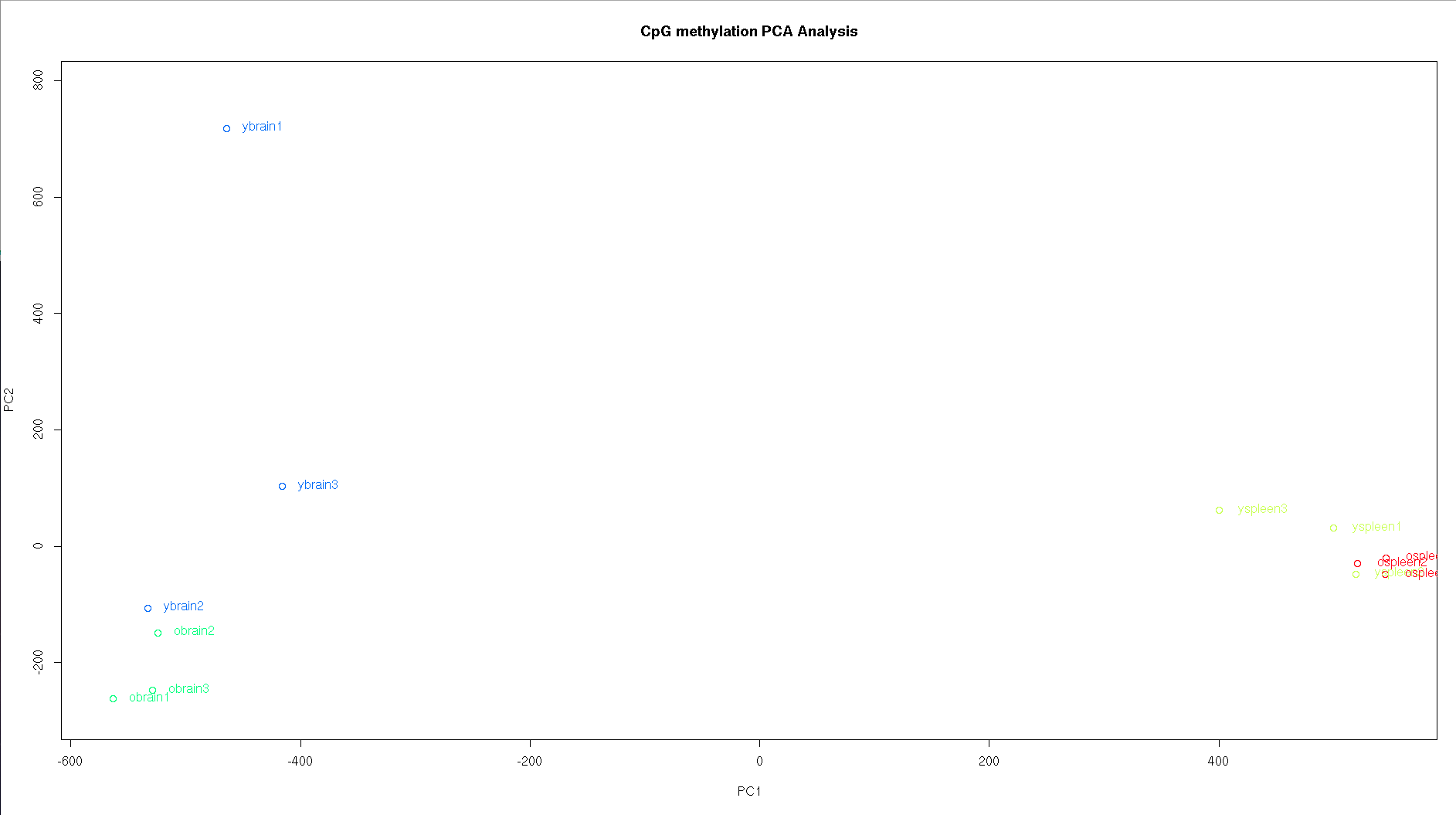
**
