# Supplementary material for: Age-specific changes in genome-wide methylation enrich for Foxa2 and estrogen receptor alpha binding sites
Source: PLoS One. 2018 Sep 26;13(9):e0203147. doi: 10.1371/journal.pone.0203147 (PMC6157835; doi:10.1371/journal.pone.0203147)

**Supplementary Figure 2 (S2 Fig).** The global methylation at CpG, CHG and CHH sites is not different between young and old specimens in either spleen or brain.


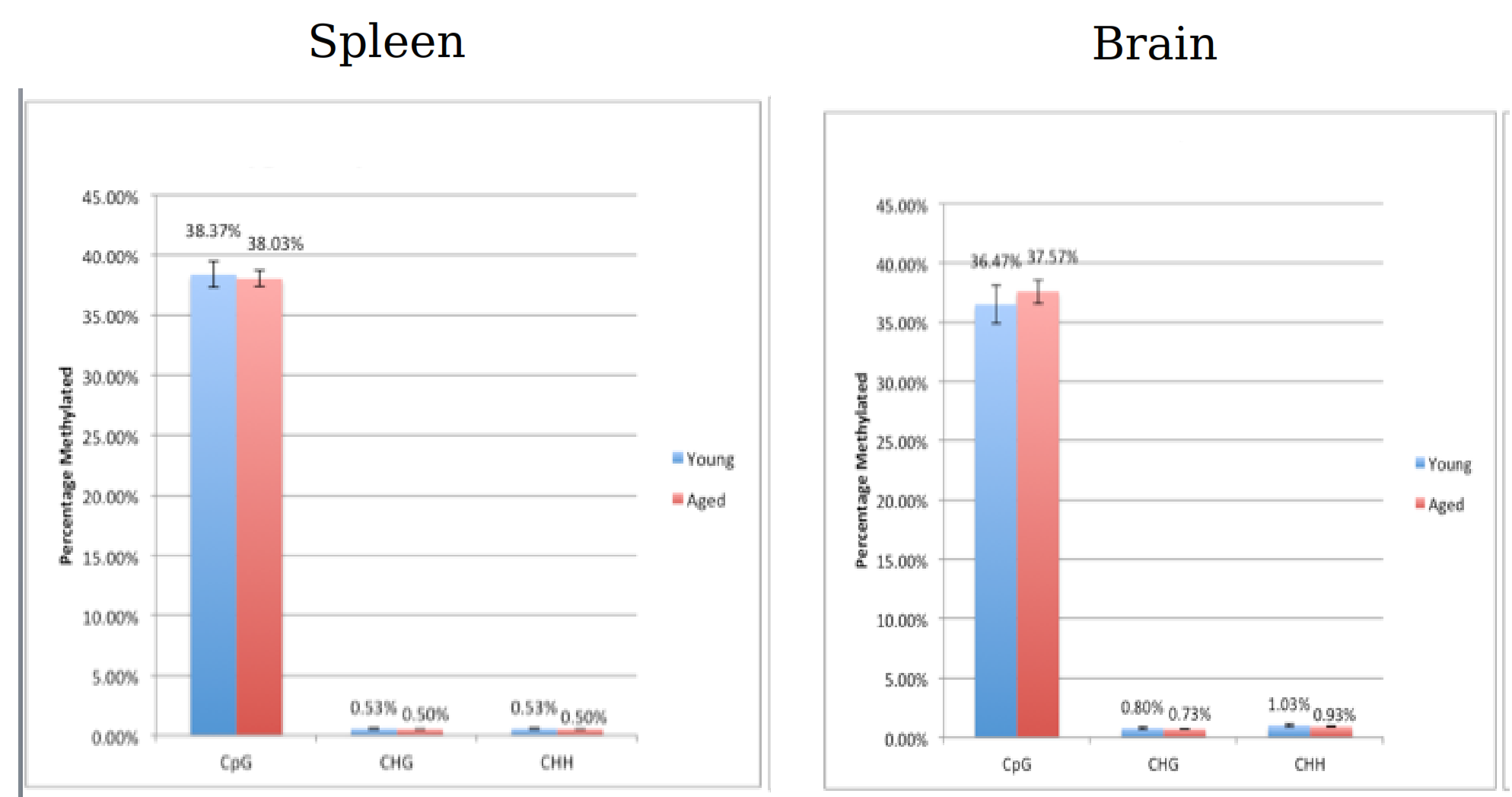

Supplement: S2 Fig — The type and percent of methylation between young and aged samples is not different across the targeted methylome. (DOCX) [file pone.0203147.s004.docx]
